# Health Disparities in the Relationship of Neighborhood Greenness to Mental Health Outcomes in 249,405 U.S. Medicare Beneficiaries

**DOI:** 10.3390/ijerph15030430

**Published:** 2018-03-01

**Authors:** Scott C. Brown, Tatiana Perrino, Joanna Lombard, Kefeng Wang, Matthew Toro, Tatjana Rundek, Carolina Marinovic Gutierrez, Chuanhui Dong, Elizabeth Plater-Zyberk, Maria I. Nardi, Jack Kardys, José Szapocznik

**Affiliations:** 1 Department of Public Health Sciences, University of Miami Miller School of Medicine, 1120 NW 14th Street, Soffer Clinical Research Center Room 1065, Miami, FL 33136, USA; tperrino@med.miami.edu (T.P.); jlombard@miami.edu (J.L.); k.wang3@med.miami.edu (K.W.); TRundek@med.miami.edu (T.R.); JSzapocz@med.miami.edu (J.S.); 2 University of Miami School of Architecture, 1223 Dickinson Drive, Building 48 Room 320G, Coral Gables, FL 33146, USA; epz@miami.edu; 3 Department of Neurology, University of Miami Miller School of Medicine, 1120 NW 14th Street, Soffer Clinical Research Center Room 1348, Miami, FL 33136, USA; CGutierrez2@med.miami.edu (C.M.G.); CDong@med.miami.edu (C.D.); 4 ASU Library, Map and Geospatial Hub, Arizona State University, Tempe, AZ 85281, USA; Matthew.Toro@asu.edu; 5 Miami-Dade County Parks, Recreation and Open Spaces Department (MDPROS), 275 NW 2nd Street, Hickman Building, 3rd floor, Miami, FL 33128, USA; mnardi@miamidade.gov (M.I.N.); jkardys@miamidade.gov (J.K.)

**Keywords:** neighborhood greenness, health disparities, neighborhood income, mental health, Alzheimer’s disease, depression, U.S. Medicare beneficiaries, older adults

## Abstract

Prior studies suggest that exposure to the natural environment may be important for optimal mental health. The present study examines the association between block-level greenness (vegetative presence) and mental health outcomes, in a population-based sample of 249,405 U.S. Medicare beneficiaries aged ≥65 years living in Miami-Dade County, Florida, USA, whose location did not change from 2010 to 2011. Multilevel analyses examined relationships between greenness, as measured by mean Normalized Difference Vegetation Index from satellite imagery at the Census block level, and each of two mental health outcomes; Alzheimer’s disease and depression, respectively, after statistically adjusting for age, gender, race/ethnicity, and neighborhood income level of the individuals. Higher block-level greenness was linked to better mental health outcomes: There was a reduced risk of Alzheimer’s disease (by 18%) and depression (by 28%) for beneficiaries living in blocks that were 1 SD above the mean for greenness, as compared to blocks that were 1 SD below the mean. Planned post-hoc analyses revealed that higher levels of greenness were associated with even greater mental health benefits in low-income neighborhoods: An increase in greenness from 1 SD below to 1 SD above the mean was associated with 37% lower odds of depression in low-income neighborhoods, compared to 27% and 21% lower odds of depression in medium- and high-income neighborhoods, respectively. Greenness may be effective in promoting mental health in older adults, particularly in low-income neighborhoods, possibly as a result of the increased opportunities for physical activity, social interaction, or stress mitigation.

## 1. Introduction

There is a growing awareness in the medical community, as well as the health and policy sectors, of the importance of addressing mental health at both the individual and population levels [1,2]. Alzheimer’s disease, the most common form of dementia, is on the rise, owing to the rapid aging of the population, both in the United States and globally [3]. In addition to being a leading cause of disability and poor health, Alzheimer’s disease is now the sixth-leading cause of death in the United States [4], and together with other dementias, is the seventh-leading cause of death worldwide [5]. At the same time, depression is one of the most common mental health disorders, and is of increasing concern both in the United States and globally [6,7], with the World Health Organization reporting that depression is the single largest contributor to disability in the world [6].

Neighborhood built (physical) and social environmental characteristics are increasingly recognized as key determinants of health, including physical health outcomes such as obesity [8,9,10], and mental health outcomes such as depression [11,12,13] and cognitive functioning [14,15,16]. More specifically, neighborhood greenness (vegetative presence) has recently been identified as a relatively novel correlate of physical health outcomes [17,18,19,20,21], as well as mental health outcomes such as depression and cognitive functioning [12,16], by providing an environment more amenable to physical activity as well as social interaction, and possibly also stress mitigation [12,22,23,24]. In our prior work, we found that neighborhood greenness was related to lower risk for chronic diseases in U.S. Medicare beneficiaries, with evidence suggesting that these greenness-to-health relationships are strongest for low-income beneficiaries, who may be most vulnerable to their local conditions, because they may lack the resources to escape the influences of their immediate neighborhood [17]. (See also, e.g., Mitchell & Popham [25] and Maas et al. [26] for similar patterns of results.) It has been suggested that the lack of greenness in many low-income neighborhoods may be a pathway toward deleterious health impacts in residents of low-income neighborhoods, by limiting opportunities for the physical activity and positive social interactions that may occur in parks, green spaces, tree-lined sidewalks and gathering places [12,27,28]. The present study seeks to examine the relationship of neighborhood greenness to two leading mental health outcomes—Alzheimer’s disease and depression—in a population-based sample of U.S. Medicare beneficiaries, and to discern whether the strength of greenness-to-mental health relationships varies by neighborhood income level.

## 2. Materials and Methods

### 2.1. Study Design

The present study is part of a larger study examining the relationship of the neighborhood built environment in relation to chronic health conditions among a population-based sample of 249,405 Medicare beneficiaries aged ≥65 years in Miami-Dade County, Florida, USA, whose location did not change from 2010 to 2011 [17]. Miami-Dade County is the seventh-largest county by population in the United States, with 2.7 million residents, and is the largest county by population in the Southeastern United States [29,30]. The present analyses focus on the relationship of neighborhood greenness (mean Normalized Difference Vegetation Index (NDVI)) for each resident’s Census block to each of two mental health outcomes: Alzheimer’s disease and depression. This comparison was further analyzed in relation to neighborhood income levels.

### 2.2. Data

The U.S. Centers for Medicare and Medicaid Services’ (CMS’) Master Beneficiary Summary File (MBSF), which provides the age, gender, race/ethnicity, annual data on health outcomes [31], and location of each beneficiary, was obtained for all of Miami-Dade County, Florida, for the calendar years 2010–2011 (available through www.resdac.org). The Chronic Conditions Segment of the CMS MSBF includes information on 27 chronic conditions, based on CMS’ algorithms for each chronic condition, using all of a beneficiary’s claims in each calendar year [32]. The current analyses focused on the presence/absence of two mental health outcomes—Alzheimer’s disease and depression, respectively—from the CMS’ Chronic Conditions Segment, using CMS’ chronic conditions algorithms for diagnoses of each of these mental health outcomes for the 2011 calendar year [32]. These chronic conditions algorithms are available on the CMS’ Chronic Conditions Data Warehouse website (https://www.ccwdata.org/web/guest/condition-categories) [32].

Greenness (i.e., vegetative presence) was assessed using the U.S. National Aeronautics and Space Administration’s (NASA) Advanced Spaceborne Thermal Emission and Reflection Radiometer (ASTER) satellite imagery. A 15 × 15 m spatial resolution enables calculation of NDVI at the Census block level. NDVI ranges from −1 to +1, with higher values indicating greater greenness or presence of healthy, dense vegetation [12,17,18,19,33,34,35,36]. We derived the mean NDVI for all 36,563 Miami-Dade County Census blocks for 2011 [17]. The unit of analysis for the relationship between NDVI and health was a change of 0.1 in NDVI. Previous work indicates that a change of 0.1 corresponds to meaningful and discernible changes in urban and suburban land use [17,34].

Neighborhood median household income was obtained at the Census block group level from 2011 U.S. Census Bureau data. As in previous work, we used three levels of neighborhood median household income to stratify the sample based on the distribution of neighborhood income across all Census block groups in the County: lowest income quartile (≤$31,600); middle two income quartiles (≥25th to ≤75th percentile); and highest income quartile (≥$62,400) [17,34].

Several steps were used to derive the final sample, as described in detail by Brown et al. [17]. The Miami-Dade County 2011 CMS Master Beneficiary Summary File had 407,296 unique Medicare beneficiaries. To obtain the final sample, the following beneficiaries were excluded: (1) 11,507 who lived outside Miami-Dade County; (2) 14,296 who had died; (3) 3572 who had end-stage renal disease; (4) 64,109 who were younger than 65 years or born prior to 1900; (5) 14,401 for whom the Geolytics software could not match to a specific Census block; (6) 12,132 who belonged to an racial/ethnic group representing <1% of the County’s population aged over 65 years; (7) 34,584 who moved during the period 2010–2011; and (8) 3650 who lived in a nursing home for all or part of the year. The final cohort included 249,405 Medicare beneficiaries, aged 65 years or older, who lived at one location for the period 2010–2011. Census blocks were determined using Geolytics ZIP+4 software, which converts each ZIP code plus 4-digit extension to a Census block, block group, and tract [37]. The University of Miami’s Human Subjects Research Office (FWA00002247, ePROST Protocol#20110948) and CMS’ Privacy Board (approval granted under CMS DUA# 24971) approved this study.

### 2.3. Statistical Analyses

SAS version 9.3 (SAS Institute Inc., Cary, NC, USA) was used to conduct descriptive and multilevel analyses. PROC GENMOD was used to conduct the three-level multilevel analyses. A three-level framework was used for the main analyses. The Level 3 variable was neighborhood median household income, obtained at the Census block group level from the 2011 U.S. Census data. The Level 2 variable was greenness, obtained for all 36,563 Census blocks in the County using the mean block level NDVI. Level 1 individual variables were race, ethnicity, age, and gender. To handle multilevel regression for binary variables, for each of the two dichotomous mental health outcomes—the absence/presence of a diagnosis of Alzheimer’s disease and depression—PROC GENMOD was used with a binary response distribution.

Descriptive statistics are presented in Table 1. The study sample characteristics were similar to those of adults aged 65 years and older living in Miami-Dade County, with 58% female, 13% black, 19% non-Hispanic white and 68% Hispanic residents [38]. Greenness NDVI scores were on average lower in lower-income neighborhoods than in higher-income neighborhoods; however, within each income quartile, there was a relatively large range of NDVI scores. Lower-income neighborhoods’ residents were more likely to have Alzheimer’s disease and depression diagnoses, and on average were more likely to be a racial/ethnic minority (Hispanic or black), female and older, when compared to residents of medium- or high-income neighborhoods.

## 3. Results

Higher levels of greenness as measured by mean NDVI were associated with a notable reduction in each of the two mental health outcomes: Each increase of 0.1 in NDVI was associated with statistically significantly lower odds of Alzheimer’s disease (by 10%) and depression (by 15%). Table 2 presents the main analyses on the relationship of greenness with each health outcome for the overall sample.

There was a reduced risk of Alzheimer’s disease (by 18%) and depression (by 28%) for elders living in Census blocks that were 1 SD above the NDVI mean, as compared to elders living 1 SD below the mean.

Further analyses examined whether neighborhood income level moderated the relationship of greenness with each mental health outcome. For Alzheimer’s disease, the cross-level interaction term between NDVI and neighborhood income level failed to attain statistical significance (*p* = 0.0652). Analyses stratified by neighborhood income level revealed that each increase of 0.1 in NDVI was associated with lower odds of Alzheimer’s disease for residents of low- (OR = 0.871, *p* < 0.0001), medium- (OR = 0.905, *p* < 0.0001), and high-income neighborhoods (OR = 0.925, *p* = 0.0061).

Analyses for depression showed that the cross-level interaction between neighborhood income and greenness was statistically significant (*p* = 0.0002). Higher levels of NDVI corresponded to greater mental health benefits for residents of lower-income neighborhoods, as compared to higher-income neighborhoods: For each increase of 0.1 in NDVI, there were lower odds of depression for beneficiaries living in low- (OR = 0.790, *p* < 0.0001) and medium-income neighborhoods (OR = 0.847, *p* < 0.0001), than in high-income neighborhoods (OR = 0.883, *p* < 0.0001).

## 4. Discussion

Higher levels of greenness (as measured by mean NDVI at the Census block level) were associated with reduced odds of two mental health outcomes: Alzheimer’s disease and depression. These findings are consistent with prior research showing that higher levels of greenness, assessed by mean NDVI, were related to lower levels of body mass index (BMI) [34], overweightness and obesity [19,39]; fewer obesity-related chronic conditions [17]; and greater physical activity [40]; as well as lower levels of symptomatology for depression, anxiety, and stress [12]. This study builds on prior findings suggesting that higher levels of greenness or access to residential green space may reduce depressive symptomatology [12,41], and potentially enhance cognitive functioning [15,16]. However, to our knowledge, this is the first study to have linked neighborhood greenness to Alzheimer’s disease, in any population.

Reductions in likelihood of Alzheimer’s disease and depression were associated with relatively small changes in mean block level NDVI. In fact, a change of 0.178 units in mean NDVI (equivalent to the difference between 1 SD above the mean and 1 SD below the mean for greenness) was associated with notable reductions in Alzheimer’s disease diagnoses (18%) and depression diagnoses (28%). Because the present results are correlational, the findings can only be suggestive. However, it would appear that small improvements in greenness or vegetative presence can beneficially impact residents’ mental health. In South Florida’s warm climate, more tree-canopy and shaded streets may increase time spent outdoors, and opportunities for physical activity and social interaction [39,42]. More greenness may also mitigate stress through the (hypothesized) restorative effects of nature exposure [22,23]. It has been noted elsewhere that physical activity and social interaction may reduce the risk of depression [43,44], as well as dementia [45,46]. Moreover, nature exposure has been linked to reductions in rumination, which may be beneficial for mental health [47]. Furthermore, it has even been suggested that vegetative presence may potentially increase exposure to the microbiome, including beneficial microorganisms, which may have health (and possibly mental health) benefits for residents, potentially by decreasing the risk of inflammation-induced psychiatric conditions and their symptom severity [48].

Stronger mental health benefits were associated with high levels of neighborhood greenness for residents of low-income neighborhoods, compared to residents of medium- and high-income neighborhoods. We found that, all other things being equal, an increase of 0.1 in NDVI was associated with an even greater reduction (of 21%) in odds of depression in a low-income neighborhood, as compared to a 15% reduction in a medium-income neighborhood, and a 12% reduction in a high-income neighborhood.

Increasing greenness, therefore, from 1 SD below to 1 SD above the mean was associated with greater reductions in odds of depression in low-income neighborhoods (by 37%), compared to medium-income (27%) and high-income neighborhoods (21%), respectively. These findings are consistent with other suggestions in the literature that residents of lower-income neighborhoods may have less access to green areas than residents of higher-income neighborhoods, which may be one pathway leading to health disparities, by decreasing the opportunities for physical activity or positive social interactions that may occur in green spaces or parks [25,42]. Future research should therefore consider the role of increasing greenness (such as through the planting of shade trees) as one relatively low-cost possible intervention through which the burden of disease may be reduced for residents of lower-income neighborhoods; however, more research is needed to identify the most effective interventions that may best improve the mental and physical health and well-being of residents in areas lacking in tree canopy or green space [49].

### Limitations

The generalizability of these findings is yet to be determined. This study needs to be replicated across populations and locales. To partially address selection bias, we excluded individuals who moved during the period 2010–2011. However, this does not address persons who moved prior to 2010, nor their reasons for moving (which is not provided by Medicare data). We further recognize that the etiology and time course of Alzheimer’s disease and depression are quite different, and that this result requires replication as well as the elucidation of mechanisms, including other biological, behavioral, or environmental variables as mediators and moderators of greenness to mental health relationships. Medicare claims data do not provide information on potential mediating variables such as physical activity, social interactions, air/noise pollution, or attention restoration [16,22,23,50]. Moreover, potential moderators such as perceived safety or aesthetics and genetic risk profiles (e.g., apolipoprotein E as a risk factor for Alzheimer’s disease) were not available [51], nor were individual-level confounders such as smoking and individual socioeconomic status. Moreover, the NDVI does not reveal types of greenery or vegetation. Future research is needed that addresses the specific types and quality of greenness and green areas, some of which may be more accessible to recreation and socialization than others. For example, issues of density, safety, access, and views of green areas may all be important for mental wellbeing [52,53,54]. The role of public/accessible versus private/inaccessible green areas is incredibly important for policy and interventions, as is access to local parks and green spaces with well-maintained infrastructure (e.g., benches and paths). We acknowledge that more-affluent versus less-affluent neighborhoods may vary in their access and/or proximity to high-quality public green spaces, which may account in part for the stronger benefits of greenness for mental health in lower-income neighborhoods, as reported in this paper. Studies are therefore needed with more rigorous research designs, such as longitudinal prospective cohort studies, that can examine the specific types and quality of vegetation and green spaces, the effects of moves, and reasons for moves; all of which may give us a clearer understanding of the basis for these greenness-to-health relationships, and aid in developing possible interventions for the most underserved populations (e.g., tree-planting and increasing access to well-maintained parks in low-income neighborhoods) [55,56,57].

## 5. Conclusions

In a population-based sample of 249,405 U.S. Medicare beneficiaries, higher levels of block-level greenness (mean NDVI) are associated with reduced odds of each of two mental health outcomes: Alzheimer’s disease and depression, respectively. Importantly, because all the participants were U.S. Medicare beneficiaries, they theoretically had similar access to health care. Most importantly, the greenness-to-mental health relationships were generally stronger and more consistently positive (at least for depression) in lower-income neighborhoods, as compared to higher-income neighborhoods. These findings are consistent with prior research suggesting that nature exposure, opportunities for socializing, walking, or stress mitigation (which may result from greenness or vegetation) may contribute to better physical and mental wellbeing in older adults [58,59,60]. The present results suggest that, particularly in low-income neighborhoods, increasing greenness may reduce the likelihood of depression and dementia at the population level, thereby enhancing elders’ quality of life.

## Figures and Tables

**Table 1:** Descriptive statistics for the overall sample, and by neighborhood income level.

Variable	Overall Sample (All Neighborhood Income Levels)				Low-Income Neighborhoods (Lowest Quartile on Income)				Medium-Income Neighborhoods (Middle 50% on income)				High-Income Neighborhoods (Highest Quartile on Income)				F (X^2^) Test	*p*-Value
	N (%)	Mean	(SD)	Range	N (%)	Mean	(SD)	Range	N (%)	Mean	(SD)	Range	N (%)	Mean	(SD)	Range		
N (Beneficiaries)	249,405	---	---	---	62,383	---	---	---	124,898	---	---	---	62,124	---	---	---	---	---
	(100.0%)				(25.01%)				(50.08%)				(24.91%)					
Neighborhood Median Household Income ^a^	---	51.4	(30.7)	0.0–250.0	---	23.0	(6.0)	0.0–31.6	---	45.3	(8.9)	31.7–62.4	---	92.4	(31.9)	62.4–250	263,465 *	<0.0001
Main Predictor: NDVI ^b^	---	−0.02	(0.09)	−0.40–0.43	---	−0.06	(0.08)	−0.40–0.40	---	−0.03	(0.08)	−0.24–0.38	---	0.03	(0.10)	−0.28–0.43	17,425.4 *	<0.0001
Individual Demographics																		
Age	---	76.33	(7.50)	65–111	---	76.79	(7.51)	65–111	---	76.34	(7.48)	65–111	---	75.85	(7.50)	65–111	244.6 *	<0.0001
Gender (% Female)	58.33%	---	---	---	59.07%	---	---	---	58.88%	---	---	---	56.48%				(116.8 *)	<0.0001
Race/Ethnicity (RTI) ^c^																	(21.647 *)	<0.0001
Hispanic (%)	65.56%	---	---	---	73.74%	---	---	---	68.48%	---	---	---	51.48%	---	---	---		
Non Hispanic White (%)	23.29%	---	---	---	9.78%	---	---	---	20.27%	---	---	---	42.92%	---	---	---		
Black (%)	11.15%	---	---	---	16.48%	---	---	---	11.26%	---	---	---	5.60%	---	---	---		
Outcome Variables:																		
Alzheimer’s Disease Dx (%) ^d^	5.49%	---	---	---	7.26%	---	---	---	5.17%	---	---	---	4.34%	---	---	---	(562.1 *)	<0.0001
Depression Dx (%) ^d^	9.25%	---	---	---	13.01%	---	---	---	8.41%	---	---	---	7.18%	---	---	---	(1475 *)	<0.0001

Abbreviations: Dx = Diagnosis; NDVI = Normalized Difference Vegetation Index; SD = Standard Deviation; * = *p* < 0.0001. ^a^ Neighborhood income for the Census block-group, in thousands of dollars [17,34]. ^b^ NDVI assessed by greenness/vegetative presence at the Census block level, with a possible range from −1 to +1 [34]. ^c^ Beneficiary-level race/ethnicity reported by CMS [31]. ^d^ Diagnoses of Alzheimer’s disease and depression, respectively, from CMS’ 2011 Chronic Conditions Segment [32].

**Table 2:** NDVI relationships to health outcomes for the overall sample, and by neighborhood income level.

Health Outcome Variables (Models)	Overall Sample (All Neighborhood Income Levels, N = 249,405) ^a^				Low-Income Neighborhoods (Lowest Quartile on Income, N = 62,383)				Medium-Income Neighborhoods (Middle 50% on Income, N = 124,898)				High-Income Neighborhoods (Highest Quartile on Income, N = 62,124)				F-Test of NDVI ^b^ X Neighborhood Income Interaction	
Individual Diagnoses (Binary Logit Models)	b	SE	*p*-Value	OR (95% CI)	b	SE	*p*-Value	OR (95% CI)	b	SE	*p*-Value	OR (95% CI)	b	SE	*p*-Value	OR (95% CI)	F-Value	*p*-Value
Alzheimer’s Disease Dx ^c^	−0.0855	0.0102	<0.0001 *	0.901 (0.870, 0.932)	−0.1380	0.0337	<0.0001 *	0.871 (0.815, 0.931)	−0.0997	0.0240	<0.0001 *	0.905 (0.863, 0.949)	−0.0777	0.0283	0.0061 ^†^	0.925 (0.875, 0.978)	F = 5.46	0.0652
Depression Dx ^c^	−0.1686	0.0140	<0.0001 *	0.845 (0.822, 0.868)	−0.2353	0.0318	<0.0001 *	0.790 (0.743, 0.841)	−0.1665	0.0195	<0.0001 *	0.847 (0.815, 0.880)	−0.1244	0.0215	<0.0001 *	0.883 (0.847, 0.921)	F = 16.67	0.0002 ^‡^

Abbreviations: b = Estimate; CI = Confidence Interval; Dx = Diagnosis; N = number of beneficiaries; NDVI = Normalized Difference Vegetation Index; OR = Odds Ratio; SE = Standard Error. * = *p* < 0.0001; ^†^ = *p* < 0.01; ^‡^ = *p* < 0.001. ^a^ Neighborhood income for the Census block-group, in thousands of dollars [17,34]. ^b^ NDVI assessed by greenness/vegetative presence at the Census block level, with a possible range from −1 to +1 [34]. ^c^ Diagnoses of Alzheimer’s disease and depression, respectively, from CMS’ 2011 Chronic Conditions Segment [32].
